# Expression and Role of Colony Stimulating Factor 1 Receptor During Odontogenesis

**DOI:** 10.3390/jdb14020023

**Published:** 2026-05-18

**Authors:** Ashina Nagra, Ling-Yi Chen, Soheil Saeidiborojeni, Jessica M. Rosin, Siddharth R. Vora

**Affiliations:** 1 Department of Oral Health Sciences, Faculty of Dentistry, University of British Columbia, Vancouver, BC V6T 1Z3, Canada; ashina.nagra@ubc.ca (A.N.); lingyi.chen@alumni.ubc.ca (L.-Y.C.); soheilsd@student.ubc.ca (S.S.); 2 Department of Oral Biological and Medical Sciences, Faculty of Dentistry, University of British Columbia, Vancouver, BC V6T 1Z3, Canada; jessica.rosin@dentistry.ubc.ca

**Keywords:** odontogenesis, CSF1R, osteoclasts, bone resorption, tooth morphogenesis, tooth-bone interface

## Abstract

In osteopetrotic mice with homozygous inactivating mutations in the colony stimulating factor 1 (*Csf1^op/op^*) or its receptor (*Csf1r^−/−^*) gene, teeth fail to erupt due to severe reduction in osteoclastogenesis. Dental abnormalities have been described in the unerupted teeth of these models, but it remains unclear whether these defects arise from direct roles of CSF1R in odontogenesis or indirectly from impaired bone remodeling associated with failed eruption. Here, we examined the spatiotemporal expression of CSF1R during tooth development and inhibited CSF1R pharmacologically in utero using PLX5622 during early stages of tooth morphogenesis. Teeth and surrounding bone were analyzed at embryonic and postnatal stages using histology and high-resolution micro-computed tomography. Embryonic CSF1R inhibition resulted in reproducible abnormalities in incisor and molar morphology that were evident before and after birth and were associated with loss of normal bone remodeling at the tooth–bone interface. In contrast, postnatal CSF1R inhibition did not affect the structure or continuous growth of adult incisors. Together, these findings demonstrate a temporally restricted, indirect role for CSF1R in odontogenesis that is independent of tooth eruption and associated with remodeling of the bony crypts surrounding developing teeth by CSF1R-dependent cells.

## 1. Introduction

Tooth development proceeds through a series of reciprocal interactions between ectoderm-derived oral epithelium and neural-crest-derived ectomesenchyme, ultimately generating the mineralized tissues of the tooth [1,2,3]. These epithelial–mesenchymal interactions control tooth number, position, size, and crown morphology through several well-conserved signaling pathways [4,5,6]. Mammalian evolution has trended toward fewer teeth and increasingly complex crown morphologies supporting specific functional demands during mastication [7,8,9,10]. In mice, crown morphogenesis occurs from ~E13.5 to birth, progressing through the bud, cap, and bell stages under the direction of enamel knots and associated signaling centers [2,6].

The dental follicle and the tooth–bone interface (TBI) are well-established regulators of alveolar bone remodeling and tooth eruption, coordinating osteoclast and osteoblast activity around developing teeth. Bone remodeling at the TBI creates space within the crypt for tooth germ expansion and is essential for eruption-related axial movement [11,12,13]. However, how this surrounding tissue influences the shaping of the tooth germ during embryonic crown morphogenesis remains incompletely defined.

**Figure 1:**
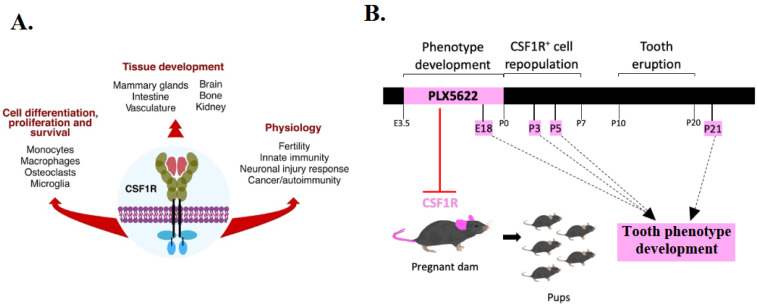
Roles of CSF1R (**A**) and experimental design used in study (**B**). Pregnant dams received CSF1R inhibitor PLX5622 via diet from E3.5 to birth (P0). Offspring were collected at different prenatal and postnatal timepoints (pink highlights) to assess the effects of in utero CSF1R inhibition on tooth development.

Colony stimulating factor 1 receptor (CSF1R) is a receptor tyrosine kinase expressed on monocyte/macrophage-lineage cells, including osteoclast precursors, microglia, and tissue macrophages, and regulates their proliferation, differentiation, and survival [14,15,16,17,18]. Osteoclast precursors require CSF1R signaling for activation and fusion into multinucleated osteoclasts, the primary effectors of bone resorption [19,20]. CSF1R also regulates the trophic functions of microglia and macrophages in the growth and development of the brain, mammary gland, vasculature, pancreas, and intestine [21] (Figure 1A). Since CSF1R signaling is important for multiple macrophage-lineage populations, perturbation of this pathway may have broader effects on the developing microenvironment.

Because developing teeth sit within bony crypts, remodeling at the TBI by CSF1R-dependent cells, including osteoclast lineage cells, is required to maintain the TBI and create space for tooth morphogenesis and eventual eruption [6,11,22]. In osteopetrotic mouse models with inactivating *Csf1* or *Csf1r* mutations (*Csf1^op/op^* and *Csf1r^−/−^*), osteoclastogenesis fails, leading to dense bone, unerupted teeth, and craniofacial abnormalities [23]. Unerupted teeth in these mice show malformed crowns, reduced enamel thickness, disorganized ameloblasts and odontoblasts, and distorted roots [24,25,26]. These defects are generally attributed to eruption failure rather than a primary defect in odontogenesis [23]. However, eruption in mice occurs postnatally (P10–P20), long after crown morphogenesis is complete. Therefore, crown defects present at birth in *Csf1^op/op^* and *Csf1r^−/−^* mice raise the possibility that CSF1/CSF1R influences early odontogenesis independently of eruption.

We previously reported that pharmacologic CSF1R inhibition in utero using PLX5622 permits tooth eruption but results in mandibular and cranioskeletal size and shape abnormalities, as well as altered tooth crown morphology at postnatal stages (P21 and P28) [27,28]. In this study, we further explore the role of CSF1R during odontogenesis by defining the spatiotemporal localization of CSF1R during tooth development and examining tooth shape abnormalities resulting from in utero CSF1R inhibition. We also consider the role of osteoclast function in the ectomesenchyme surrounding the developing tooth germs as a potential mechanism underlying these phenotypes. Collectively, our findings support a model in which CSF1R regulates tooth morphogenesis indirectly by influencing remodeling of the bony crypts at the tooth–bone interface through CSF1R-dependent cells rather than acting directly on dental epithelium or odontoblast lineages.

## 2. Materials and Methods

### 2.1. Animals

CD1 mice (Charles River) were used for all experiments. Animal work was carried out in accordance with guidelines and regulations of the Canadian Council of Animal Care and received prior approval from the University of Calgary’s (protocol AC17-0191) and University of British Columbia’s (protocol A15-0202, A19-0221) animal care committees. For expression analyses, timed mated dams were sacrificed at embryonic days 13.5 (E13.5), E15.5, E16.5, and E18, and offspring were collected and processed for immunofluorescence. For embryonic CSF1R inhibition, PLX5622 (Plexxikon, San Francisco, CA, USA), was administered to pregnant dams daily via diet, from E3.5 to birth (1200 ppm added to chow AIN-76A, Research Diets, Inc., New Brunswick, NJ, USA), while control dams received control diet (AIN-76A). Considering the role of endometrial macrophages in pregnancy, drug treatment earlier than E3.5 may have interfered with implantation [29]. For some experiments, offspring were collected and sacrificed at postnatal days 3 (P3), P5, P21, and P28 (Figure 1B). P28 mice had been weaned at P21, and wet diet was provided as previously described [27]. For postnatal inhibition of CSF1R, PLX5622 was administered daily for four weeks, starting at P28 via chow, while control mice received a control diet.

### 2.2. Tissue Preparation

Whole heads were fixed in 2% or 4% paraformaldehyde for 24 h. Heads were immediately washed in PBS at 4 °C or decalcified (for specimens older than E18) in regular changes of 14% EDTA (pH 7.4) for one week before washing. Samples were processed for paraffin embedding, and 7 μm thick sections were cut along the sagittal or coronal planes for histology, histochemistry, and immunofluorescence.

### 2.3. Histology

Sections were stained with hematoxylin and eosin, Von Kossa (with MacNeal’s tetrachrome counterstain), or Pollak’s trichrome. Von Kossa staining was performed on non-decalcified sections using 5% silver nitrate under ultraviolet light, followed by development in sodium carbonate–formaldehyde and fixation in Farmer’s Diminisher before MacNeal’s tetrachrome counterstaining [30]. Pollak’s trichrome staining was performed on decalcified, hematoxylin-stained sections using a one-step dye application and rapid differentiation with 0.2% glacial acetic acid. Slides were imaged using a 3DHISTECH Pannoramic^®^ MIDI scanner (3DHISTECH Kft., Budapest, Hungary) and processed in CaseViewer (3DHISTECH Kft., Budapest, Hungary).

### 2.4. Histochemistry

Tartrate-resistant acid phosphatase (TRAP) is an established marker for osteoclast activity. Deparaffinized sections were serially rehydrated and incubated in pre-warmed TRAP staining solution containing TRAP basic incubation mix, Fast Red Violet LB salt (Sigma-Aldrich, St. Louis, MO, USA), and Naphthol AS-MX phosphate substrate (Sigma) for 1 h or until the stain had developed. They were counterstained with 0.08% fast green, air-dried, and imaged. Sections from control and PLX6522 animals were mounted on the same slide to ensure consistency in processing.

### 2.5. Immunofluorescence

Paraffin sections underwent antigen retrieval (Diva Decloaker, DV2004 Biocare Medical, Pacheco, CA, USA), were blocked in 10% donkey serum (0.5% PBT, 1 h), and incubated overnight at 4 °C with sheep anti-mouse CSF1R (10 μg/mL; R&D Systems, Minneapolis, MN, USA; Cat# AF3818; RRID:AB_884158) with or without goat anti-mouse CSF1 (10 μg/mL, R&D Cat # AF416, RRID:AB_355351). Negative controls used pre-immune sheep IgG and/or goat IgG at matched concentrations. The next day, sections were incubated with donkey anti-sheep Alexa 647 (1:250, Thermo Fisher Scientific, Waltham, MA, USA, Cat# A21448; RRID:AB_10374882) and/or donkey anti-goat Alexa 488 (1:250, Thermo Fisher, Cat# A-11055, RRID:AB_2534102) for 1 h, counterstained with Hoechst 33342 (1:5000, Thermo Fisher), and imaged using a 3DHISTECH Pannoramic^®^ MIDI scanner (100 ms exposure; focus frequency of 5–10). DAPI, 488, and Cy5 filter sets were used.

### 2.6. Μicro-Computed Tomography (μCT)

Fixed mouse heads were embedded in 0.5% agarose and scanned on a Scanco μCT100 (Scanco Medical, Brüttisellen, Switzerland; 55 kVp, 200 μA; 34.4 μm voxel size). DICOMs were imported into 3D Slicer (Slicer.org) for tooth segmentation using consistent thresholding across samples. Segmented volumes were converted to 3D models, and fiducial landmarks were placed on homologous tooth surfaces. X, Y, and Z coordinates were extracted and Euclidean distances calculated to obtain length and width dimensions. Repeat landmarking yielded ICC values > 0.88. Selected scans were also visualized in Drishti (V2.6.4, ANU VizLab, Australian National University, Canberra, Australia) using clipping planes to assess internal structure, with enamel rendered translucent via intensity-based transform functions to expose the dentin–enamel junction.

### 2.7. Sample Size and Statistics

Animals were obtained from multiple independent litters at each developmental stage and distributed across experimental modalities (IHC, histology, TRAP, µCT, and morphometric analyses), such that not all animals were used for every assay, and we tried to ensure that more than one litter contributed to each assay. Sample sizes for each experiment and timepoint, including the number of contributing litters, are summarized in Supplemental Table S1 and noted in each figure legend. Unless otherwise specified, each animal was treated as one biological replicate. For molar dimensional analyses, both right and left molars were measured. Quantitative data are presented as mean ± standard deviation (SD). Statistical analyses were performed using unpaired two-tailed Student’s *t*-tests for comparisons between the control and PLX5622-treated groups. A *p*-value < 0.05 was considered statistically significant.

## 3. Results

### 3.1. CSF1 Receptor and Ligand Expression in Developing Teeth

**Figure 2:**
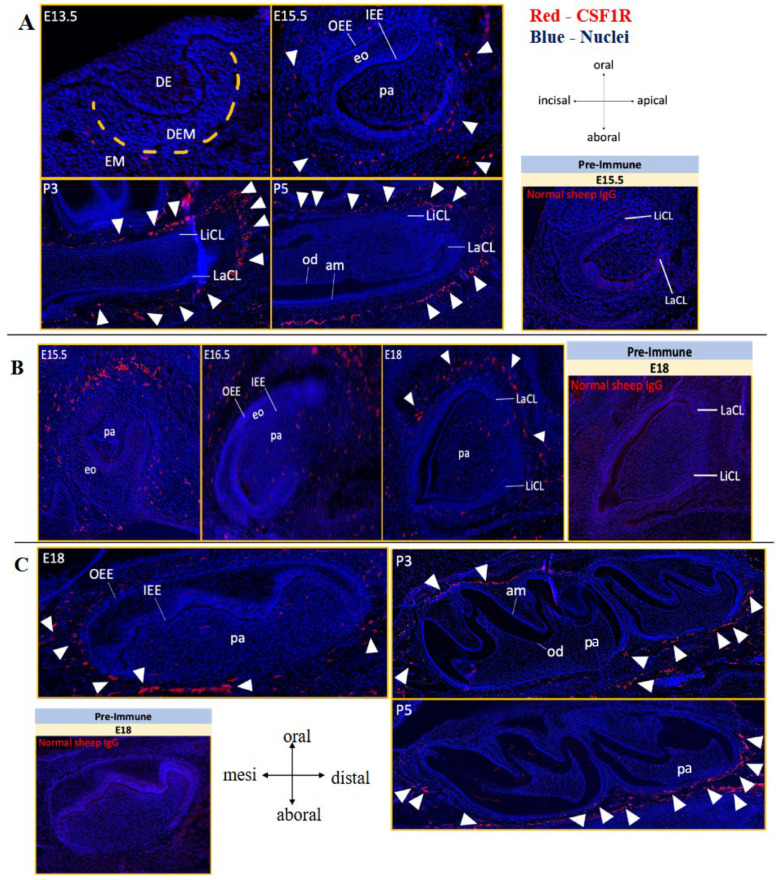
CSF1R expression during odontogenesis. CSF1R and nuclei in red (arrowheads) and blue, respectively. (**A**) Mandibular incisor germs at E13.5 (condensing dental ectomesenchyme is outlined in yellow lines), E15.5, P3, and P5. Pre-immune negative control at E15.5 on right. (**B**) Maxillary incisor germs at E15.5, E16.5, and E18. Pre-immune negative control at E18 on right. (**C**) Mandibular molar germs at E18, P3, and P5. Pre-immune negative control at E18 on right. n = 2–3 animals analyzed per stage [see Supplemental Table S1 for details]. Ameloblasts (am), dental epithelium (DE), dental ectomesenchyme (DEM), dental papilla (pa), ectomesenchyme (EM), enamel organ (eo), inner enamel epithelium (IEE), labial cervical loop (LaCL), lingual cervical loop (LiCL), odontoblasts (od), outer enamel epithelium (OEE).

**Figure 3:**
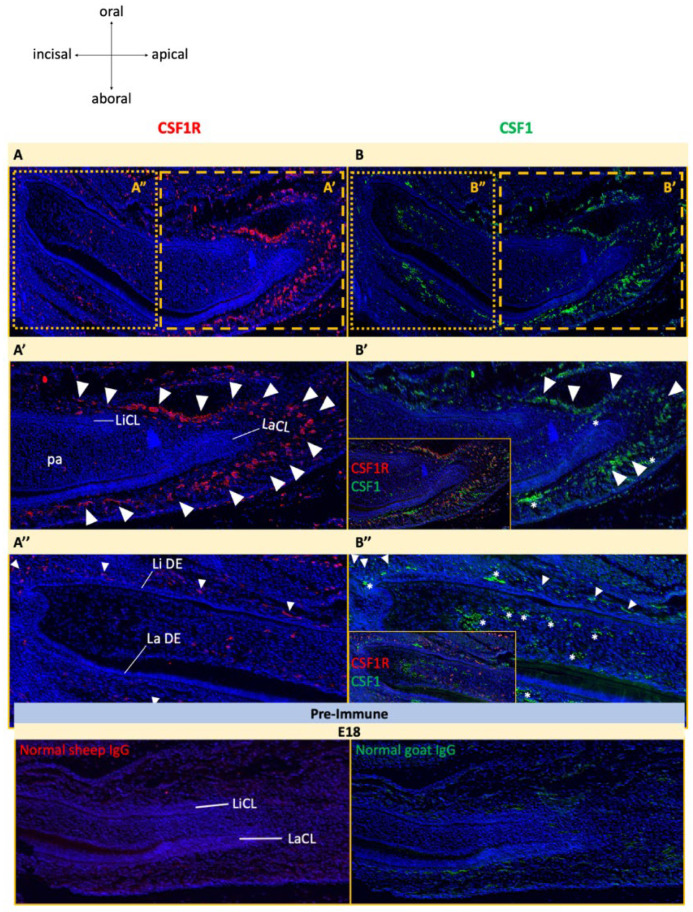
CSF1/CSF1R expression in the E18 mandibular incisor. (**A**) CSF1R stained in red and (**B**) CSF1 stained in green, with nuclei stained blue. Magnified views of the apical ends (**A′**,**B′**) and incisal ends (**A″**,**B″**) from A and B. Insets in (**B′**,**B″**) show CSF1/CSF1R double immunostaining. Autofluorescent blood cells are marked by asterisks (*) while CSF1- and CSF1R-expressing cells are marked with arrowheads. Pre-immune negative controls at E18 on bottom panel. n = 3 animals [see Supplemental Table S1 for details]. Dental papilla (dp), labial cervical loop (LaCL), labial dental epithelium (La DE), lingual cervical loop (LiCL), lingual dental epithelium (Li DE).

Immunostaining for CSF1R was examined across key stages of odontogenesis. At E13.5, CSF1R expression was largely absent from the incisor bud (Figure 2A). From E15.5 onward, CSF1R-positive cells increased within the ectomesenchyme surrounding the outer enamel epithelium (OEE) of incisors and molars, with stronger staining near the apical ends of the tooth germs compared to the incisal ends (Figure 2A–C). Staining was also more pronounced labially than lingually (Figure 2B). At E18, double immunofluorescence showed that CSF1 exhibited a similar ectomesenchymal distribution to CSF1R, with prominent co-localization near the apical cervical loop region (Figure 3B–B″). Both proteins showed faint staining within the dental papilla (Figure 3A–B″). No CSF1R immunostaining was detected in any dental epithelium-derived tissues, including OEE, stellate reticulum, stratum intermedium, inner enamel epithelium, ameloblasts, or in odontoblasts and their precursors at either embryonic or early postnatal stages (Figure 2 and Figure 3). Similarly, cells at the continuously growing apical cervical loops of incisors did not express CSF1R.

### 3.2. Dental Phenotype Resulting from Embryonic CSF1R Inhibition

We inhibited CSF1R in utero by administering PLX5622 to pregnant dams between E3.5 and birth (Figure 1B) [27,28]. Immunostaining at E18 confirmed loss of CSF1R around developing teeth in PLX5622-exposed embryos, consistent with depletion of CSF1R-dependent cell populations within the surrounding ectomesenchyme, which include osteoclast/macrophage lineage cells (Supplemental Figure S1).

**Figure 4:**
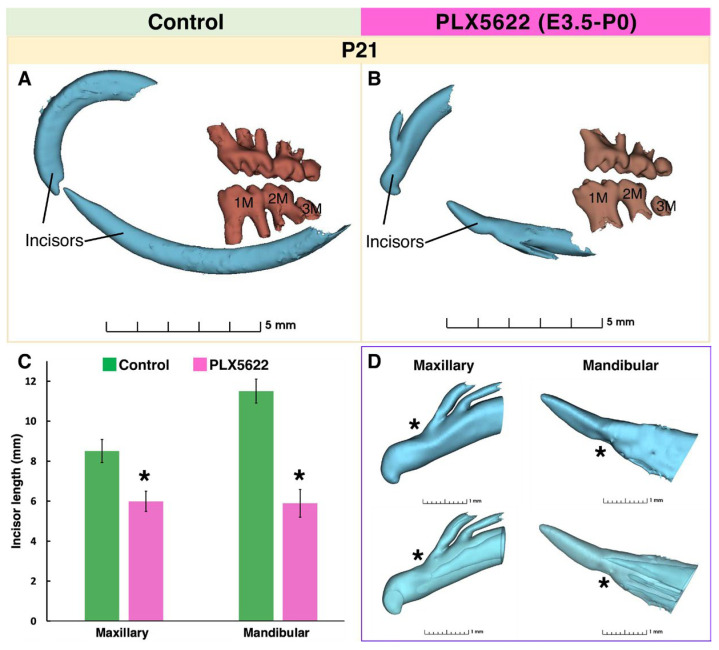
μCT reconstruction of mouse dentition at P21 of control (**A**) and PLX5622 exposed mice (**B**). The apical end of the mandibular incisor in CSF1R-inhibited mice, fails to elongate past the first molar (**B**) as compared to controls (**A**). Incisor length quantified from μCT reconstruction (**C**) with control animals’ teeth in green and PLX5622 exposed animals’ teeth in pink [mean ± SD, n = 5 animals per group, * = *p* < 0.05, unpaired two-tailed *t*-test]. Maxillary and mandibular incisors in PLX5622-exposed animals shows notching on the labial surface (*, (**D**)). Top row shows surface reconstruction, and bottom row shows teeth with translucent surface, so that the inner outline of the pulpal areas is visible. Representative images shown. µCT datasets were obtained from n = 7–8 animals per group [see Supplemental Table S1 for details]. First molar (1M), second molar (2M), third molar (3M).

**Figure 5:**
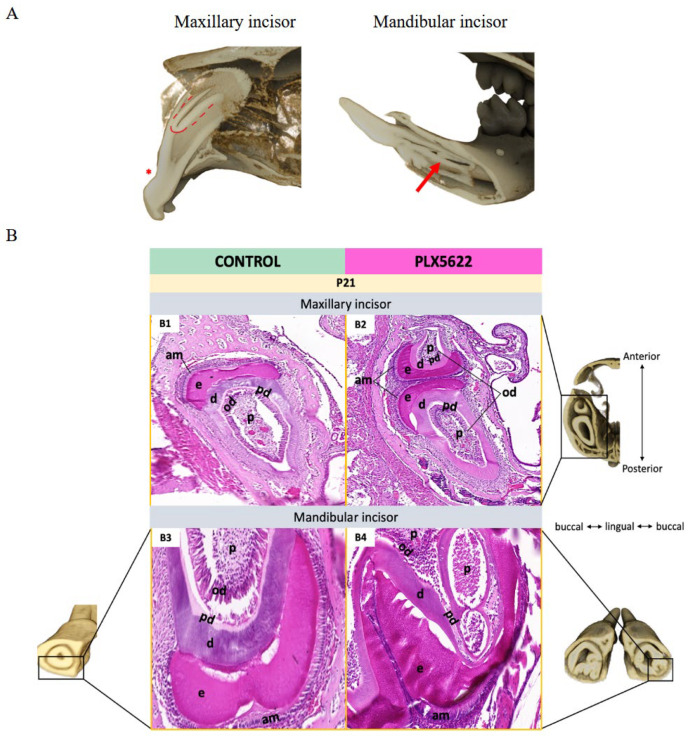
Incisor phenotypes in CSF1R-inhibited mice. μCT imaging showing sagittal section through the maxillary and mandibular incisors in CSF1R-inhibited mice ((**A**), right and left respectively). The maxillary incisor is branched (dotted red line) on its facial surface, apical to the notched deformation (*, (**A**)), while the mandibular incisor shows infolding of mineralized tissue at the apical part (red arrow, (**A**)). (**B**) Left: Maxillary and mandibular incisors in control mice stained with H&E (**B1**,**B3**). Right: Maxillary and mandibular incisors in CSF1R-inhibited mice stained with H&E (**B2**,**B4**). Duplicated tooth phenotype can be seen in (**B2**) (and adjacent axial uCT section). This ectopic, branched structure with dental cells, enamel, dentin, and pulp is flipped and attached to original incisor (**B2**). Invagination of the dentin–enamel junction with grossly disorganized dentin and pulp can be noted in mandibular incisors (**B4**). n = 3 animals per group [see Supplemental Table S1 for details]. Ameloblasts (am), dentin (d), enamel (e), odontoblasts (od), predentin (pd), pulp (p).

At P21, μCT, and histology showed marked defects in the incisors of PLX5622-exposed pups. Both maxillary and mandibular incisors were shorter than controls (Figure 4A–C). Mandibular incisors failed to extend posterior to the first molar roots, whereas in controls they reached beyond the third molars (Figure 4A,B). A consistent labial notching was present in all incisors at approximately 1.0–1.5 mm from the incisal tip (Figure 4D). Maxillary incisors showed a branched, geminated-like structure apical to this notch, consisting of smaller enamel and dentin elements fused to the main tooth (Figure 5A). Histologically, these branches appeared to be comprised of enamel and dentin in a mirrored orientation to the original tooth structure (Figure 5B2). Mandibular incisors exhibited irregular infoldings at the dentin–enamel junction, pulp chamber distortion, and formation of supplemental canals (Figure 5B4). Notching, branching, and infoldings occurred in all examined PLX5622-exposed pups at P21 (n = 8), representing 100% incidence, and were not observed in control animals (n = 7, Supplemental Table S1 for details).

**Figure 6:**
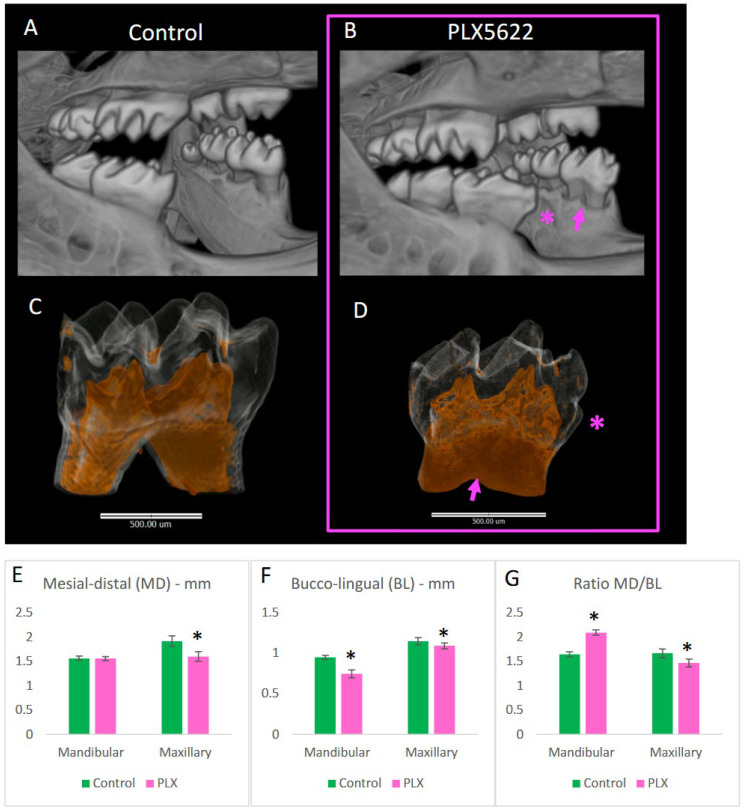
Molar phenotypes in CSF1R-inhibited mice. μCT reconstructions of molars from control (**A**,**C**) and PLX5622-exposed animals (**B**,**D**). The mandibular first molars display a distinct mesial bulge of enamel ((**B**), pink *), mirroring the dentin–enamel junction contour visible below the translucent rendering of enamel in ((**D**), pink *). The root furcation is also more apically positioned in PLX5622 exposed animals ((**B**,**D**), pink arrow) as compared to controls (**A**,**C**). The dimensions of the teeth are also altered for maxillary and mandibular teeth. n = 5–6 animals per group (10–12 molars total; bilateral measurements), mean ± SD, * = *p* < 0.05 in (**E**–**G**), unpaired two-tailed *t*-test. For dimensional analyses, each animal was treated as a biological replicate, with right and left molars measured per animal [see Supplemental Table S1 for details].

Molar teeth were also affected (Figure 6A–D). Mandibular first molars were significantly narrower in the buccolingual dimension, but had similar mesial-distal length compared to controls, resulting in an altered occlusal table shape (Figure 6E–G). Maxillary first molars were reduced in both dimensions (Figure 6E–G). Mandibular first molars showed a distinct mesial bulge, with the enamel contour mirroring the shape of the underlying dentin–enamel junction (Figure 6D). The distance from the buccal height of contour to the bifurcation was increased (mean 1.15 ± 0.07 mm vs. 1.05 ± 0.08 mm in controls, *p* = 0.033, n = 5–6 animals per group: 10–12 molars total of bilateral measurements; see Supplemental Table S1 for details), consistent with a mild taurodontic phenotype (Figure 6B,D; pink arrows). Notably, this taurodontic-like morphology was observed in all PLX5622-exposed animals at P21 (n = 8) and P28 (n = 4) and was absent in controls, with findings consistent across animals derived from multiple independent litters (see Supplemental Table S1 for details). Second and third molars appeared normal and were not examined further.

### 3.3. CSF1R Inhibition Impacts Tooth Development at Embryonic and Early Postnatal Stages

**Figure 7:**
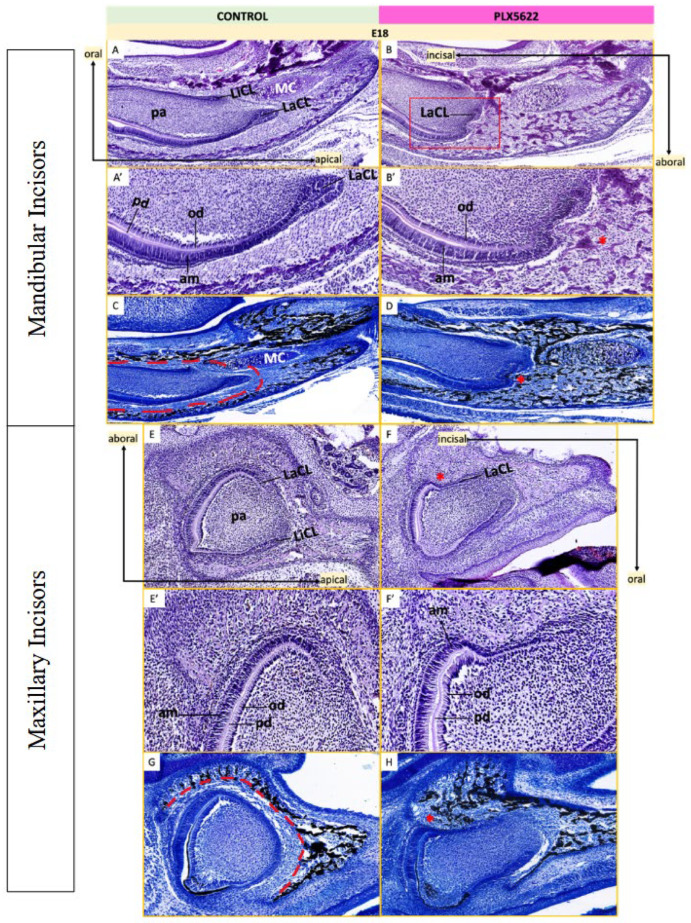
Histological analysis of incisors at E18. Left: Tooth germs in control mice stained with H&E (**A**,**E**) and Von Kossa/tetrachrome (**C**,**G**). Right: Tooth germs in mice exposed to CSF1R inhibition with PLX5622 in utero stained with H&E (**B**,**F**) and Von Kossa/tetrachrome (**D**,**H**). Both mandibular (**A**–**D**) and maxillary incisors (**E**–**H**) were examined. (**A′**,**B′**,**E′**,**F′**) show zoomed-in views of the labial cervical loop regions from (**A**), (**B**), (**C**) and (**D**), respectively. Infolding of odontogenic tissues near labial cervical loop is seen in mandibular incisors (*, (**B′**,**D**)). Red lines denote boundary of the tooth–bone interface around the developing tooth germ (**C**,**G**), which was dramatically reduced in PLX5622-exposed animals (**D**,**H**). Mineralized alveolar bone trabeculae (black staining in (**D**,**H**)) closely approximates the developing dental germ epithelium, with notching of the labial surface in maxillary teeth (*, (**F**,**H**)). n = 3–5 animals per group [see Supplemental Table S1 for details]. Ameloblasts (am), dental papilla (pa), labial cervical loop (LaCL), lingual cervical loop (LiCL), Meckel’s cartilage (MC), odontoblasts (od), predentin (pd).

**Figure 8:**
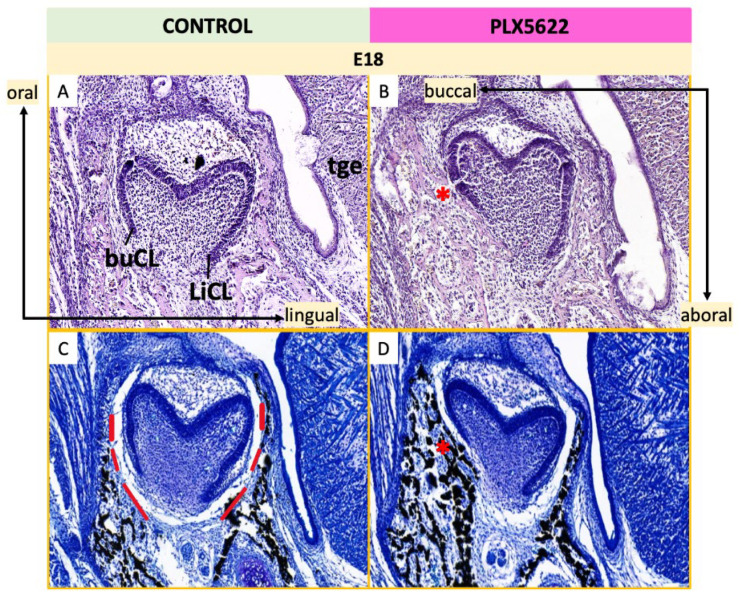
Histological analysis of mandibular first molar at E18. Coronal sections. Left: Tooth germs in control mice stained with H&E (**A**) and Von Kossa/tetrachrome (**C**). Right: Tooth germs in mice exposed to CSF1R inhibition with PLX5622 in utero stained with H&E (**B**) and Von Kossa/tetrachrome (**D**). Molar germs develop within bony crypts (black staining with dotted red line boundary in (**C**)). The alveolar bone closely contacts the buccal side of the molar germ in PLX5622-treated animals (*, (**B**,**D**)). n = 2 animals per group [see Supplemental Table S1 for details]. Buccal cervical loop (buCL), lingual cervical loop (LiCL), tongue (tge).

To investigate the origins of altered dental morphology following CSF1R inhibition, developing teeth were examined at embryonic and early postnatal stages (E18-P5). Von Kossa staining with tetrachrome counterstain was used to visualize mineralized bone surrounding the tooth germs (Figure 7). In control embryos at E18, incisor germs were enclosed within organized bony crypts and separated from surrounding bone by a distinct tooth–bone interface (TBI, Figure 7C,G). In contrast, CSF1R-inhibited embryos showed marked reduction or loss of the TBI, with direct contact between mineralized bone and both mandibular and maxillary tooth germs (Figure 7D,H). At E18, mandibular first molar germs were narrower in the buccolingual dimension in CSF1R-inhibited embryos compared to controls (Figure 8). Control molars developed within defined bony crypts separated by a clear TBI (Figure 8C, dotted red line), whereas these boundaries were largely absent following CSF1R inhibition (Figure 8D).

**Figure 9:**
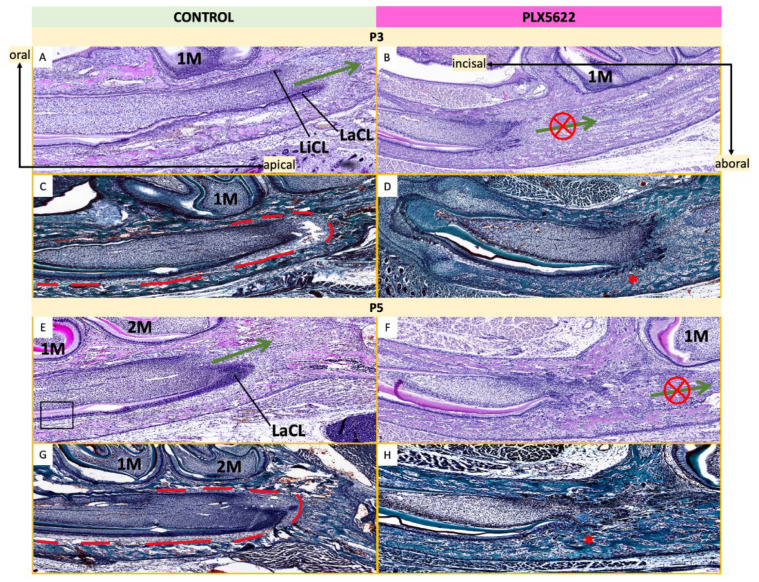
Postnatal histological analysis of mandibular incisors. Left: Tooth germs in control mice stained with H&E (**A**,**E**) and Pollak’s trichrome stain (**C**,**G**). Right: Tooth germs in mice exposed to CSF1R inhibition with PLX5622 in utero stained with H&E (**B**,**F**) and Pollak’s trichrome stain (**D**,**H**). Sections through the mandibular incisors at P3 (**A**–**D**) and P5 (**E**,**F**) are shown. In control animals (**A**,**C**,**E**,**G**) the cervical loops of the developing incisors elongated past the level of the first molar and second molar (1M and 2M respectively, green arrows). However, in PLX5622-exposed animals, the dental epithelium failed to elongate past the first molar (1M, green arrow with red “X”). At P3 and P5, soft tissue space ((**C**,**G**)-red lines) separated the developing incisor from surrounding bone (green staining). However, in PLX5622-exposed animals, bone trabeculae (*) infiltrated the soft tissue spaces and came into close contact with the developing incisor germs (**D**,**H**). n = 3–4 animals per group per age [see Supplemental Table S1 for details]. First molar (1M), second molar (2M), labial cervical loop (LaCL), lingual cervical loop (LiCL).

Dental abnormalities persisted postnatally despite withdrawal of PLX5622 at birth. At P3, mandibular incisor cervical loops in control mice were positioned posterior to the first molars (Figure 9). Pollak’s trichrome staining revealed a collagen-rich bone matrix separated from the incisor germ by a clear TBI (Figure 9C, Supplemental Figure S2A,A′). In CSF1R-inhibited mice, cervical loops were poorly defined and failed to extend up to the first molars (Figure 9B, Supplemental Figure S2B,B′). Apical regions of the odontogenic epithelium appeared interrupted by the closely approximated bone (Figure 9B,D). By P5, control incisor epithelium had elongated posterior to the second molars and remained separated from bone by a well-defined TBI (Figure 9E,G). In contrast, incisor elongation in CSF1R-inhibited mice remained stalled anterior to the first molar, with persistent epithelial contact with bone and absence of a clear TBI (Figure 9F,H).

**Figure 10:**
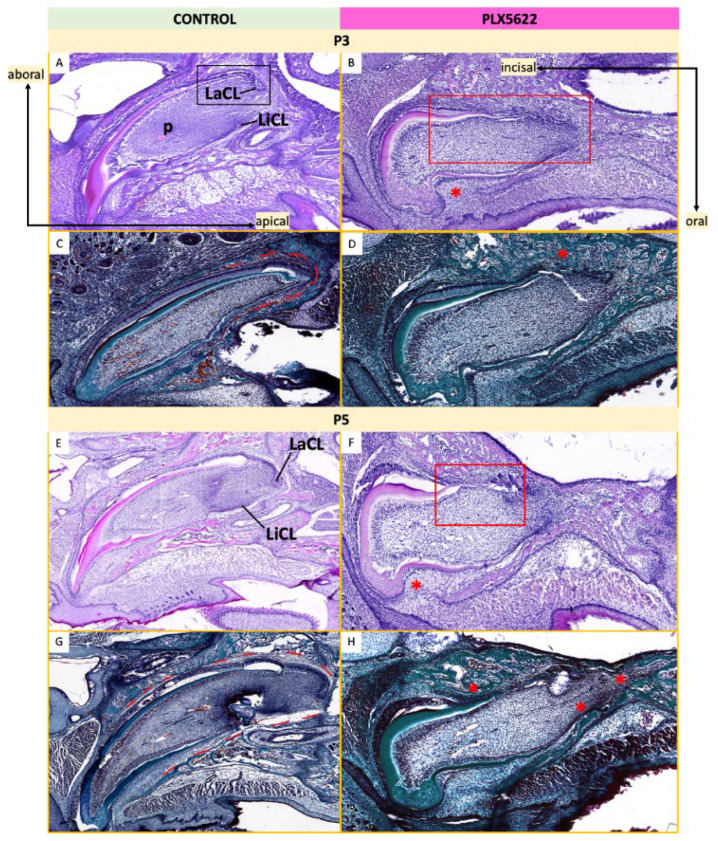
Postnatal histological analysis of maxillary incisors. Left: Tooth germs in control mice stained with H&E (**A**,**E**) and Pollak’s trichrome (**C**,**G**). Right: Tooth germs in mice exposed to CSF1R inhibition with PLX5622 in utero stained with H&E (**B**,**F**) and Pollak’s trichrome (**D**,**H**). Sections through the maxillary incisors at P3 (**A**–**D**) and P5 (**E**,**F**) are shown. In PLX5622-exposed animals, the lingual surface of the P3 tooth germ epithelium shows indentations (*) and the labial surface of the germ near the cervical loop appears wavy and discontinuous ((**B**,**F**) red boxes), as opposed to the smooth, curved cervical loops seen in controls ((**A**) black box). Control animals show soft tissue space ((**C**,**G**) red lines) which separates the tooth germ from surrounding bone (stained green), while bone trabeculae (*) appear to have infiltrated soft tissue space and come into contact with the developing incisors in PLX5622-treated animals (**D**). Similar abnormalities are seen in teeth of PLX5622-exposed animals at P5 (**F**,**H**) compared to controls (**E**,**G**). n = 3–4 animals per group per age [see Supplemental Table S1 for details]. Pulp (p), labial cervical loop (LaCL), lingual cervical loop (LiCL).

Maxillary incisors showed similar abnormalities at P3 and P5. The normal curved morphology of control maxillary incisor germs (Figure 10C,G) was lost in the CSF1R-inhibited mice (Figure 10D,H), with disruption in the architecture of the cervical loop regions due to close approximation between bone trabeculae and the dental epithelium (Figure 10D,H, red *). While ameloblasts and odontoblasts within the developing incisors and molars appeared to retain normal polarity, the morphology of the cervical loop appeared significantly disrupted (Supplementary Figures S3 and S4).

### 3.4. Altered Osteoclast Activity During Embryonic and Postnatal Stages of Odontogenesis

**Figure 11:**
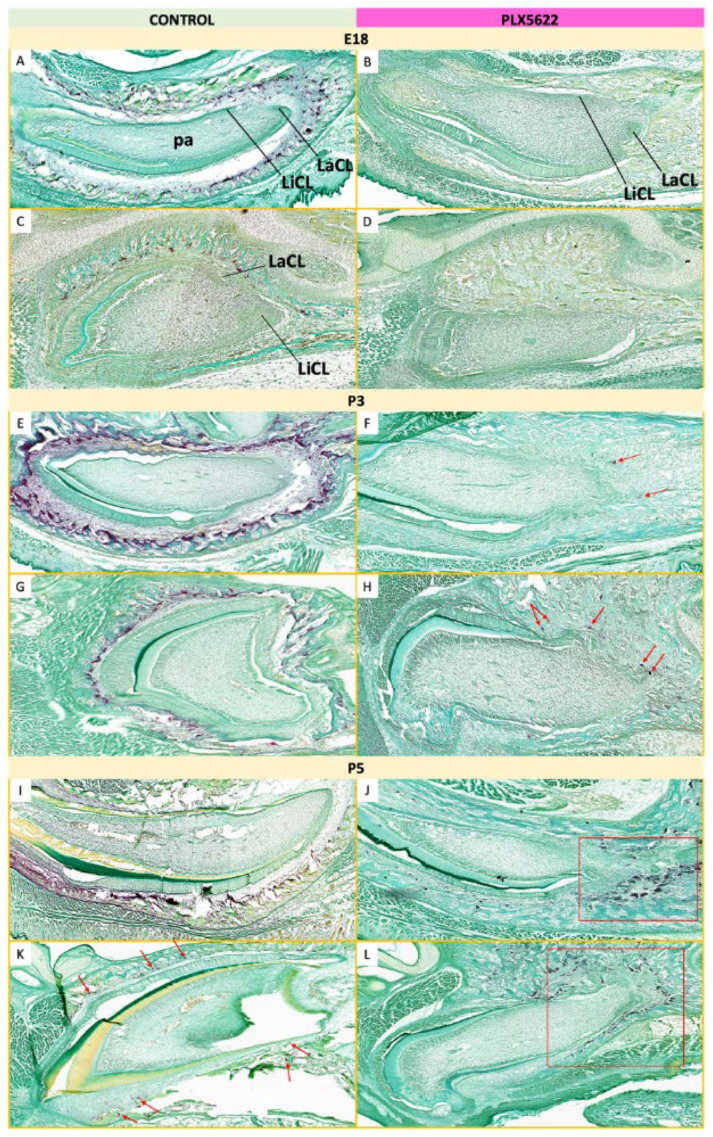
Osteoclastic activity surrounding incisors at E18, P3, and P5. TRAP^+^ cells (red/brown) line soft tissue spaces between mandibular (**A**,**E**,**I**) and maxillary (**C**,**G**,**K**) incisor germs and the surrounding bone in control mice. At E18, no TRAP^+^ cells are visible around mandibular (**B**) and maxillary (**D**) incisor germs in CSF1R-inhibited mice. At P3, a few TRAP^+^ cells are visible on the apical side of mandibular and maxillary incisor germs (red arrows in (**F**,**H**), respectively) in CSF1R-inhibited mice, with more TRAP^+^ cells repopulating the tissues surrounding mandibular and maxillary incisor germs (red boxes in (**J**,**L**), respectively), by P5. n = 3–4 animals per group per age [see Supplemental Table S1 for details]. Dental papilla (pa), labial cervical loop (LaCL), lingual cervical loop (LiCL).

Tartrate-resistant acid phosphatase (TRAP) was used to assess osteoclast activity during tooth development [31]. In control embryos at E18, TRAP-positive cells were abundant and surrounded incisor germs (Figure 11A,C). Postnatally, TRAP staining peaked at P3 and subsequently declined, with activity concentrated around the incisal regions during eruption (Figure 11E,G,I,K, Supplemental Figure S5A,A′,A″). In contrast, TRAP-positive cells were not detected around tooth germs in CSFIR-inhibited embryos at E18 (Figure 11B,D). Following withdrawal of PLX5622 at birth, osteoclasts gradually repopulated odontogenic regions. At P3, TRAP-positive cells were detectable around the apical ends of maxillary and mandibular teeth (Figure 11F,H, Supplemental Oure S5B,B′,B″), with markedly increased staining by P5 (Figure 11J,L).

### 3.5. Postnatal CSF1R Inhibition Does Not Impact Tooth Eruption and Structure

Mouse incisors grow continuously due to stem cell populations located at the cervical loops. Based on the embryonic phenotypes observed, we hypothesized that CSF1R inhibition affects tooth morphology indirectly through altered osteoclast activity at the TBI, rather than through direct effects on the odontogenic—mineralized tissue-forming cells. To test this, PLX5622 was administered postnatally starting at P28 for four weeks, followed by μCT analysis. No gross morphological differences were observed between the incisors of control and PLX5622-treated mice (Supplemental Figure S6). Cervical loop regions appeared normal, and neither branching nor infolding phenotypes were detected, indicating that postnatal CSF1R inhibition does not disrupt incisor growth or structure.

## 4. Discussion

Embryonic inhibition of CSF1R from E3.5 to birth produced highly penetrant and reproducible dental abnormalities. We used a pharmacological inhibitor rather than genetic models because *Csf1r^−/−^* mice are osteopetrotic and fail to erupt teeth [23,24,25,26], making it difficult to distinguish defects caused by failed eruption from earlier roles in odontogenesis. In contrast, transient exposure to CSF1R inhibitor PLX5622 during embryogenesis permits normal tooth eruption [27,28], allowing developmental roles of CSF1R in tooth morphogenesis to be examined independently of eruption. Our immunostaining confirmed effective suppression of CSF1R-positive cells during embryonic stages, while the recovery of TRAP activity after birth demonstrated PLX5622 reversibility and postnatal repopulation of CSF1R-positive osteoclast/macrophage lineage cells. The uniform expression of the observed tooth phenotypes suggests that temporally controlled pharmacologic inhibition provides a robust approach for interrogating developmental roles of CSF1R signaling.

Rodent incisors are specialized for gnawing, with elongated mandibular incisors and sharp chisel-like edges that occlude against maxillary incisors. This functional morphology was disrupted following embryonic CSF1R inhibition. Maxillary incisors lacked typical cutting edges and exhibited altered surface contours, suggesting abnormal wear or enamel properties. Although ameloblast and odontoblast morphology appeared largely preserved, altered enamel microstructure remains a possibility. In *op/op* mice lacking CSF1, enamel defects and reduced fracture resistance have been reported by SEM analysis [32]. Similar analyses will be required to assess enamel organization in PLX5622-exposed teeth.

A striking and reproducible phenotype in CSF1R-inhibited animals was branching of maxillary incisors. These branches consisted of enamel and dentin organized around a central canal, but with reversed enamel polarity relative to the main tooth. This phenotype differs from incisor duplication observed in Sprouty-deficient mice, where epithelial subdivision produces laterally positioned, erupted supernumerary teeth [33]. A similar duplication and branching phenotype was reported following epithelial-specific deletion of *Dicer1*, which disrupted microRNA-mediated regulation and led to expansion of the cervical loop and formation of multiple incisor axes [34]. However, in that model, branching occurred in both maxillary and mandibular incisors along the longitudinal growth axis, whereas in the present study, it was restricted to maxillary incisors and arose from the labial surface, with mandibular incisors exhibiting a distinct infolding phenotype. In contrast, branching in CSF1R-inhibited mice consistently arose from the labial surface and often occurred multiple times within a single tooth.

Mandibular incisors showed a distinct but related phenotype, with infolded dentin–enamel junctions and canal-like structures within the pulp space. Both maxillary and mandibular incisors also exhibited a characteristic bulged facial contour with a notch-like feature detectable at E18 and prominent at P21. These notches most likely reflect spatial constraints imposed by deficient bone remodeling, rather than direct effects on odontogenic differentiation. Consistent with this interpretation, regions lacking a clear TBI were observed in PLX5622-exposed embryos. Because branching and infolding phenotypes were not detectable at E18 or P3, early disruption of the TBI likely establishes abnormal spatial constraints, which later manifest as complex structural defects with continued tooth growth.

The mandibular first molar phenotype, including buccolingual narrowing and mesial contour expansion, likely reflects spatial restriction within the bony crypt during early crown morphogenesis. The absence of abnormalities in second and third molars is consistent with their later initiation, after CSF1R inhibition had ceased in our model.

Given the severity of the dental abnormalities, we initially considered whether CSF1R might act directly on odontogenic cells. Although some studies have reported *Csf1r* transcripts in ameloblasts and odontoblasts [32,35,36,37], we detected no CSF1R protein in dental epithelial or odontoblast lineages at any stage examined. The presence of *Csf1r* transcripts without detectable protein may reflect low translation efficiency or contamination of laser-captured samples with adjacent ectomesenchymal cells [32]. Instead, CSF1R and its ligand were consistently detected in ectomesenchymal tissues surrounding tooth germs. While this pattern could suggest expression by dental follicle cells, CSF1R expression is otherwise restricted to monocyte and macrophage lineages [38,39]. This supports the interpretation that CSF1R-positive macrophages and osteoclast precursors populate the follicular region during tooth development. This is reinforced by prior detection of TRAP-positive cells at the tooth bone interface and by recent single-cell transcriptomic data identifying CSF1R-positive macrophages surrounding incisor cervical loops [13,40]. During early odontogenesis, CSF1R signaling may likely mediate paracrine CSF1-dependent macrophage differentiation and osteoclast function. Further molecular characterization of odontogenic differentiation and epithelial–mesenchymal signaling pathways will be required to directly test this mechanism and is the focus of ongoing follow-up studies.

Our findings support a model in which CSF1R influences tooth morphogenesis indirectly through regulation of the TBI rather than through direct effects on odontogenic stem cells or mineralized tissue-forming cells. Although the loss of TRAP-positive cells and disruption of the TBI are consistent with impaired osteoclast-mediated remodeling, contributions from other CSF1R-positive macrophage populations cannot be excluded in this pharmacologic model. This interpretation is further supported by postnatal inhibition experiments. Continuous incisor growth depends on epithelial and mesenchymal progenitors at the cervical loop, yet postnatal CSF1R inhibition did not disrupt incisor structure or induce branching or infolding phenotypes. Therefore, CSF1R activity is not required for maintenance of dental stem cell populations in mature mice.

Instead, the timing of CSF1R inhibition appears critical. Alveolar bone development proceeds concurrently with tooth morphogenesis [22], and failure of remodeling at the TBI during embryogenesis likely results in physical encasement of incisor and first molar tooth germs by bone. When crypt expansion is restricted, odontogenic epithelium likely adapts to confined space; because odontogenic epithelium is highly responsive to spatial constraints during morphogenesis, restriction of crypt expansion may alter epithelial folding trajectories that ultimately define crown shape. Once established, these defects are not corrected after postnatal recovery of osteoclast/macrophage activity, indicating a narrow developmental window during which proper remodeling of the tooth bone interface is required. Absence of observable phenotypes in the second and third molars further support this hypothesis, since these teeth begin development at a later time, once CSF1R inhibition was reversed in our model system.

Late-returning activity of osteoclast/macrophage lineage cells may further contribute to phenotype severity. After withdrawal of PLX5622, these cells repopulate odontogenic regions, coinciding with appearance of duplication and infolding phenotypes. Release of matrix-bound growth factors by these cells could influence epithelial behavior at the cervical loop, potentially exacerbating abnormal morphogenesis at the incisors’ cervical loop. Similarly, delayed root bifurcation and taurodontic features observed here may reflect altered signaling to Hertwig’s epithelial root sheath, potentially influenced by delayed or aberrant osteoclast/macrophage activity during root initiation. However, this mechanism does not explain the striking differences between maxillary and mandibular incisor phenotypes, which remain unresolved. Similar region-specific effects have been reported in models of disrupted bone remodeling. In a mouse model of craniometaphyseal dysplasia caused by a mutation in the Ank gene, mandibular incisors exhibit abnormal cervical loop positioning and altered morphology, while maxillary incisors develop normally under the same systemic conditions, implicating the local bone environment as a key determinant of tooth phenotype [41].

PLX5622 was administered systemically to pregnant dams and may therefore have broader developmental effects beyond the craniofacial region. Previous work has shown that embryonic CSF1R inhibition impacts cranioskeletal morphology, including alterations in cranial vault shape and mandibular development, likely through effects on osteoclast-dependent bone remodeling as well as other CSF1R-dependent cell populations [28,42]. In addition, CSF1R signaling seems to be involved in the regulation of muscle development and macrophage-mediated trophic support, which may indirectly influence craniofacial form through muscle–bone interactions [43]. However, the consistent and highly localized nature of the dental phenotypes observed here, together with recovery of osteoclast activity following drug withdrawal, suggests that these effects primarily reflect transient disruption of CSF1R-dependent cell populations at the TBI rather than nonspecific systemic toxicity.

Finally, we observed sparse CSF1R-positive cells within the dental pulp. These cells are likely macrophages derived from the dental papilla, as reported previously [40,44]. While CSF1R signaling in the dental papilla could influence early cervical loop patterning, the limited and dispersed nature of pulpal staining suggests a minor contribution, relative to the surrounding follicular tissues.

## 5. Conclusions

In summary, our data indicates that CSF1R has a temporally restricted, indirect role in tooth morphogenesis that is independent of tooth eruption. Rather than acting at odontogenic epithelial or mesenchymal lineages levels, CSF1R influences remodeling at the TBI through CSF1R-dependent cells. These findings highlight the importance of the surrounding skeletal microenvironment in shaping dental morphology, and identifies CSF1R-dependent cells as key regulators during a critical window of odontogenesis. These findings have broader implications for craniofacial biology and dental development, highlighting how disruption of the skeletal microenvironment can influence tooth morphogenesis independently of intrinsic odontogenic defects. This may be relevant to developmental dental abnormalities associated with altered bone remodeling and suggests that modulation of the TBI could be an important consideration in regenerative approaches.

## Data Availability

Data supporting the findings of this study are available from the corresponding author upon reasonable request.

## Supplementary Materials

The following supporting information can be downloaded at https://www.mdpi.com/article/10.3390/jdb14020023/s1, Supplemental Table S1. Sample sizes by experimental group and developmental stage; Figure S1. PLX5622 robustness; Figure S2. Postnatal histological analysis of mandibular teeth at P3; Figure S3. Postnatal histological analysis of maxillary incisors; Figure S4. Histological analysis of mandibular first molars at P5, Figure S5. Postnatal TRAP staining of mandibular incisors at P3; Figure S6. Inhibition of CSF1R in adult mice.
