# Supplementary material for: Expression and Role of Colony Stimulating Factor 1 Receptor During Odontogenesis
Source: J Dev Biol. 2026 May 18;14(2):23. doi: 10.3390/jdb14020023 (PMC13214697; doi:10.3390/jdb14020023)
Supplement: Supplementary file 1 [file jdb-14-00023-s001.zip › jdb-4215771-supplementary.pdf]

SUPPLEMENTARY TABLES and FIGURES

Expression and Role of Colony Stimulating Factor 1 Receptor During Odontogenesis

Ashina Nagra[1], LingYi Chen[1], Soheil Saeidiborojeni[1], Jessica M. Rosin[2,3] and Siddharth R. Vora[1*]

Supplemental Table S1. Sample sizes by experimental group and developmental stage. Age indicates embryonic (E) or postnatal (P) timepoints. Values in each experimental column indicate the sample size (n) for that assay at the specified timepoint, collected from multiple litters. Experimental columns include: immunohistochemistry (IHC) of maxillary incisors, mandibular incisors, and mandibular molars; histology; TRAP staining; micro-computed tomography (μCT) phenotypic assessment; and incisor and molar dimensional analyses. Unless otherwise specified, n represents the number of animals (biological replicates). Animals were obtained from multiple litters at each stage and distributed across experimental modalities, such that not all animals were used for every assay. For μCT analyses, a subset of animals was selected for imaging, and morphometric measurements where applicable. For molar dimensional analyses, both right and left molars were measured; thus, 5-6 animals correspond to 10-12 teeth. For all other analyses, each animal contributed a single biological replicate.

Group	Age	Number of litters	Number of animals (n)								
			IHC			Histology	TRAP	μCT phenotype	Incisor dimension	Molar dimensions
			Max Inc	Mand Inc	Mand molar					
Ctrl	E13.5	1		2						
	E15.5	2	3	3						
	E16.5	2	2							
	E18	4	3		2	4	4			
	P3	3		3	2	3	3			
	P5	3		3	2	3	3			
	P21	4				3		7	5	5
	P28	3						4		
	P56	1						3		
PLX	E18	4	3	3	2	5	4			
	P3	3				4	3			
	P5	2				3	3			
	P21	4				3		8	5	6
	P28	3						4		
	P56	2						3		

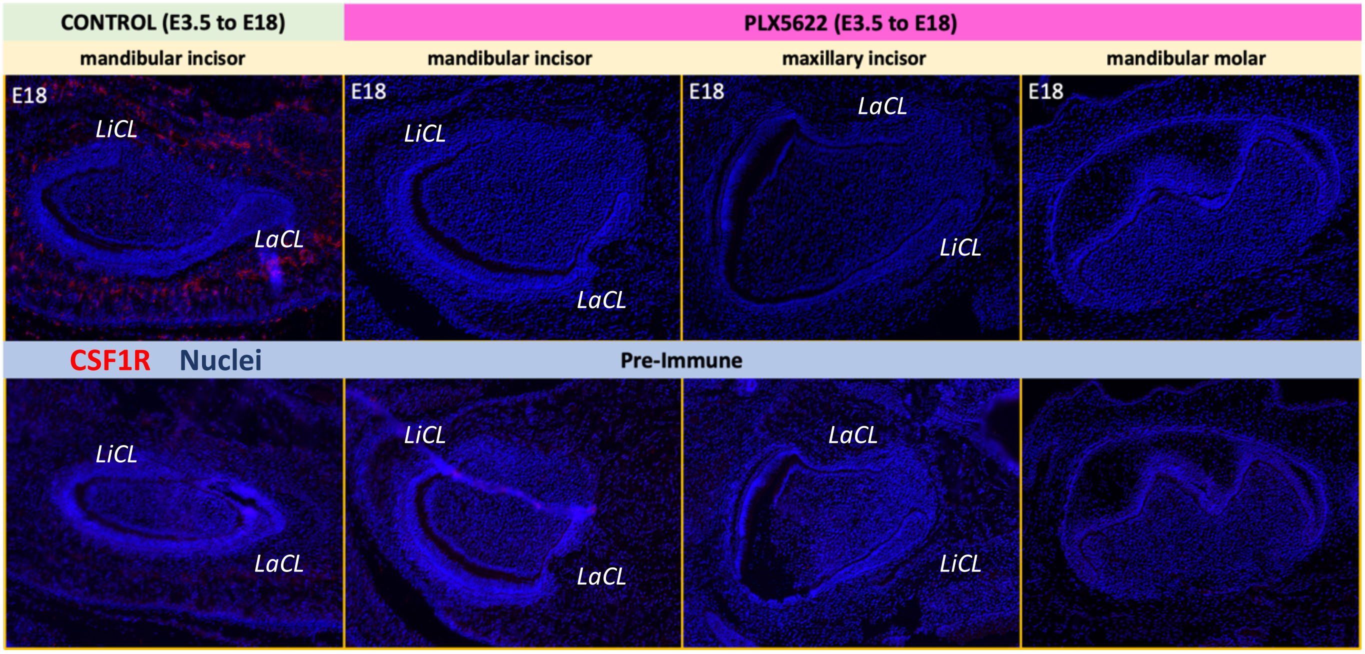

Figure S1. PLX5622 robustness. *Top row*: CSF1R immunostaining in mice exposed to control (left) or PLX5622 diet between E3.5 and E18. No CSF1R$^+$ cells are detected around tooth germs at E18 in animals exposed to PLX5622 in utero (right 3 columns), while CSF1R$^+$ cells are seen in the ectomesenchyme around the developing tooth germ in the control animals (left column and **Figs 2 and 3** in main text). *Bottom row*: pre-immune negative controls of control (left) and PLX5622 tooth germs at E18. *n* = 2-3 animals per group [See Supplemental Table 1 for details]. Labial cervical loop (LaCL), lingual cervical loop (LiCL).

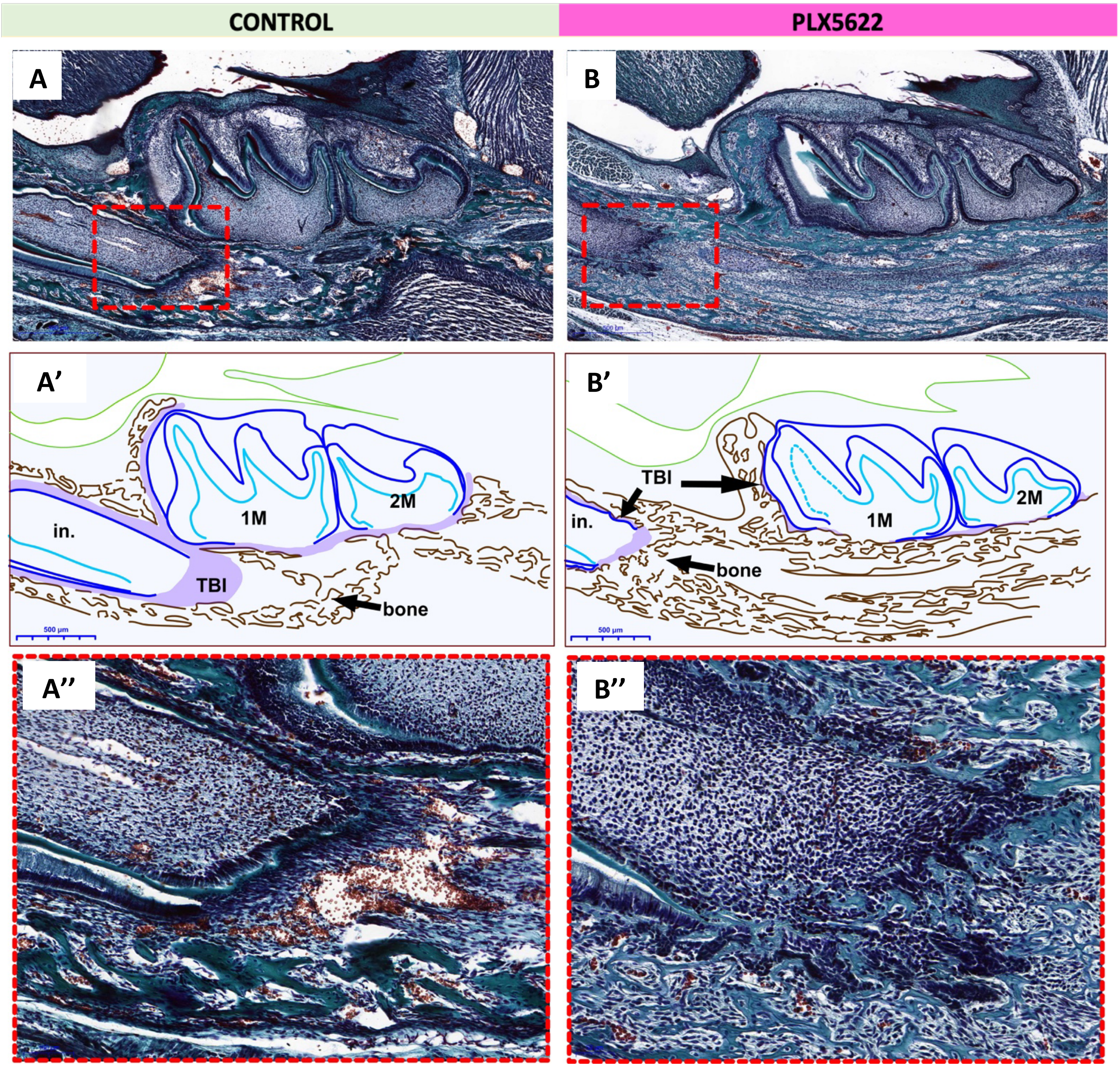

Figure S2. Postnatal histological analysis of mandibular teeth at P3 using Pollak's trichrome staining. (**A**) Control; (**B**) PLX5622-exposed. (**A′, B′)** Corresponding line drawings of (**A**) and (**B**), respectively. (**A", B")** Magnified views of (**A**) and (**B**) respectively. In the line renderings, blue lines represent the enamel organ (broken segments indicate areas of sectioning artifacts), bone boundaries are indicated in brown and the TBI is highlighted in purple. Control incisors are longer, extending to molars regions while PLX5622-exposed animals have shortened incisors. The TBI appears reduced in PLX5622-exposed animals, with bone margins extending closer to, and in some regions directly abutting, the enamel organ of the developing incisor. *n*=2 animals per group. Tooth–bone interface (TBI) first molar (1M), second molar (2M), incisor (In.).

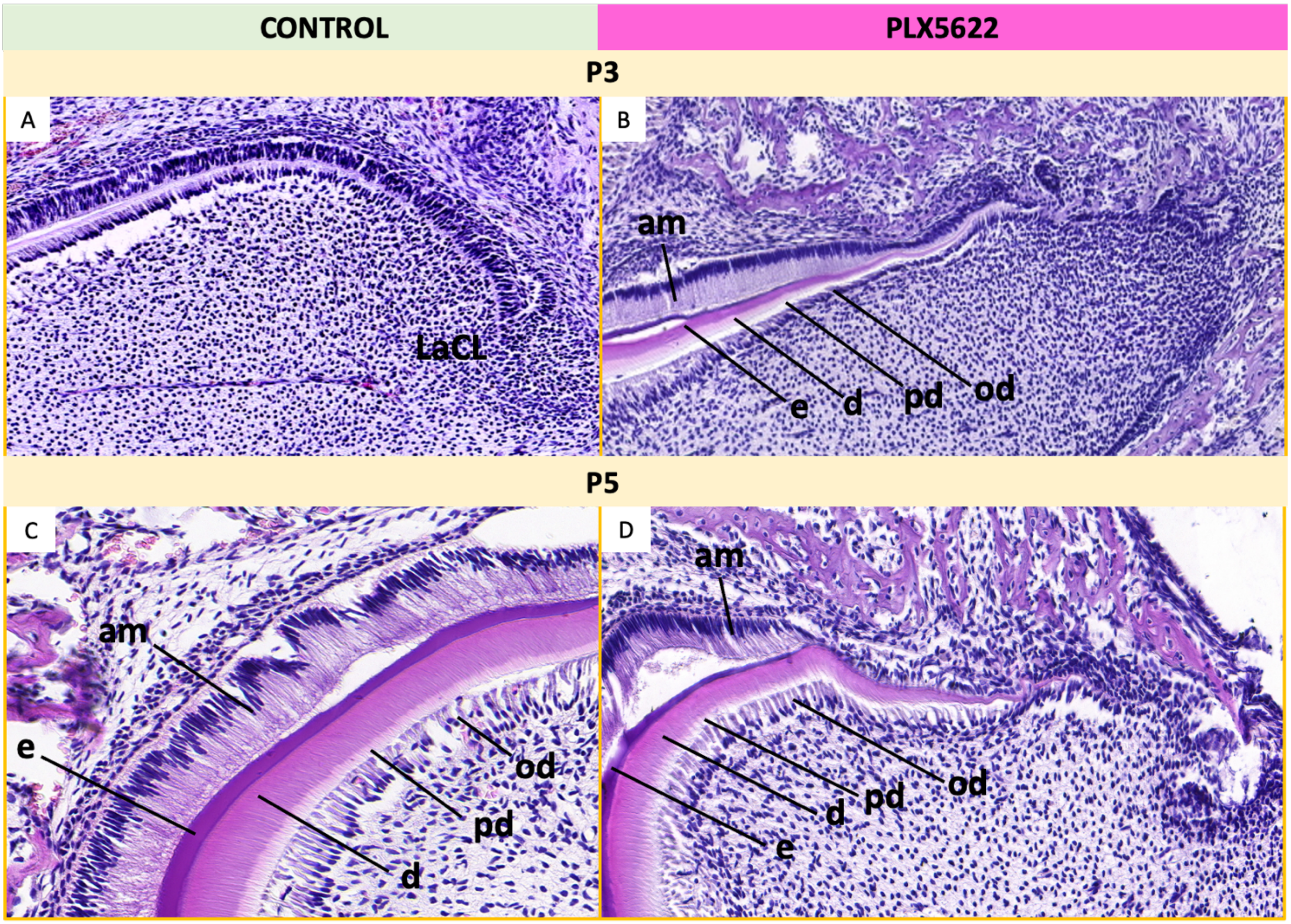

Figure S3. Postnatal histological analysis of maxillary incisors. Tooth germs in control mice (**A, C**) and mice exposed to CSF1R inhibition with PLX5622 *in utero* (**B, D**) stained using H&E at P3 (*top row*) and P5 (*bottom row*). Dental cell morphologies appear normal except at cervical loop regions. *n* = 3 - 4 animals per group [See Supplemental Table 1 for details]. Ameloblasts (am), dentin (d), enamel matrix (e), labial cervical loop (LaCL), odontoblasts (od), predentin (pd).

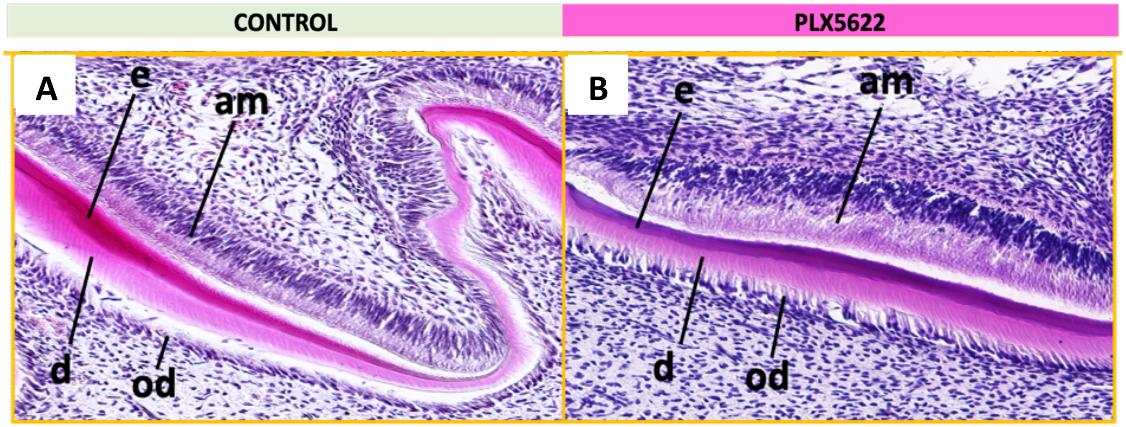

Figure S4. Histological analysis of mandibular first molars at P5. Tooth germs of control mice (**A**) CSF1R-inhibited mice (**B**) stained with H&E. n = 3 animals per group [See Supplemental Table 1 for details]. Ameloblasts (am), dentin (d), enamel matrix (e), odontoblasts (od).

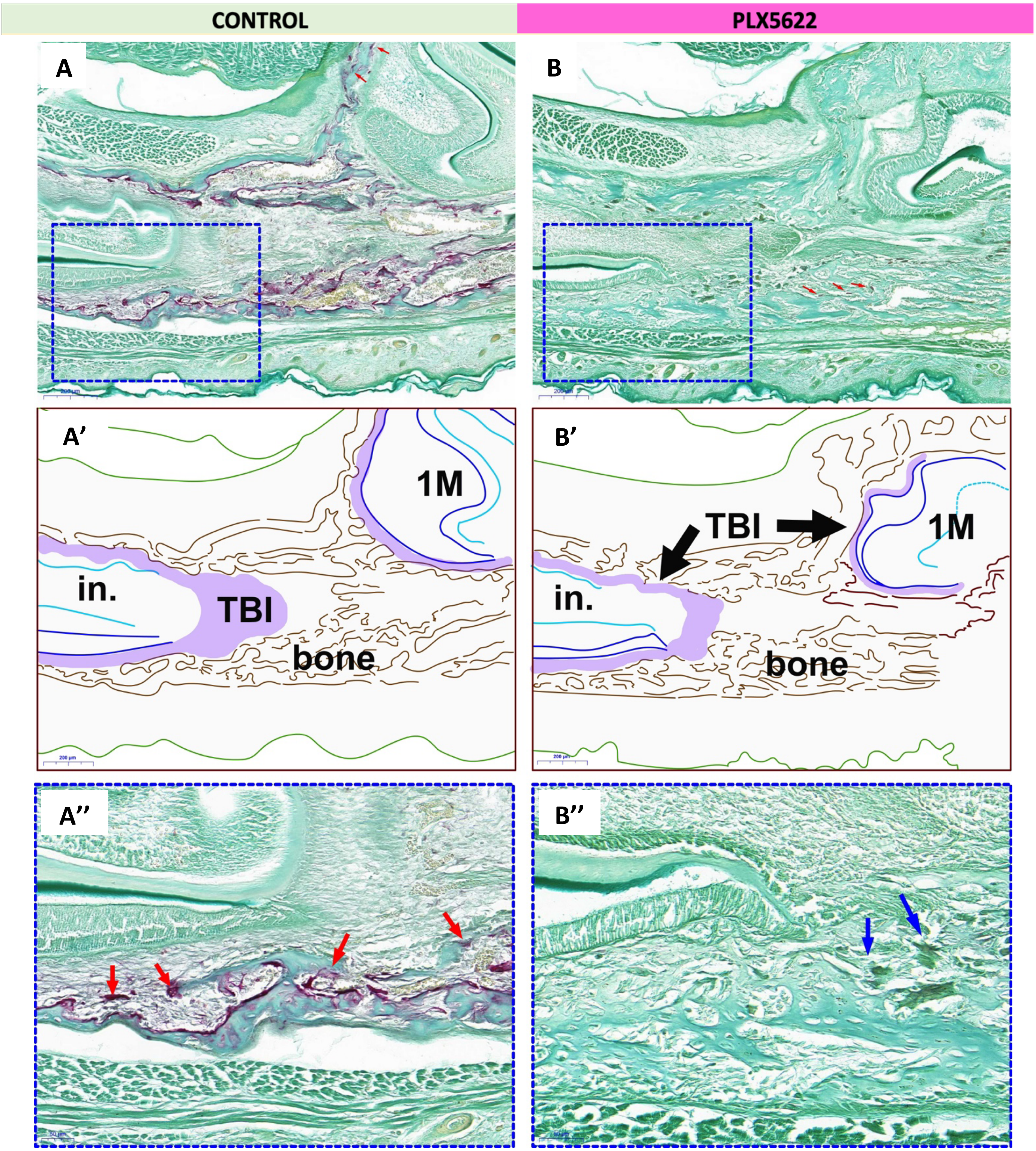

Figure S5. Postnatal TRAP staining of mandibular incisors at P3. (**A**) Control; (**B**) PLX5622-exposed. (**A′, B′)** Corresponding line drawings of (**A**) and (**B**), respectively. (**A″, B″)** Magnified views of the regions outlined by dotted blue boxes in (**A**) and (**B**). In control animals (**A, A″**), TRAP+ cells (red/brown staining; red arrows) are present along the soft tissue interface surrounding the developing incisor and adjacent bone. In PLX5622-exposed animals (**B, B″**), TRAP staining is largely absent at P3. However, cells with osteoclast-like morphology are observed adjacent to the bone surface (B" blue arrows), despite lacking TRAP positivity, suggesting early stages of osteoclast

differentiation prior to full enzymatic activity. Few TRAP positive cells are seen in B (red arrows). Magnified views highlight the lack of a clearly defined band of tissue surrounding the developing teeth TBI, in PLX5622-exposed animals. *n*=3 animals per group. Tooth–bone interface (TBI) first molar (1M), second molar (2M), incisor (In.).

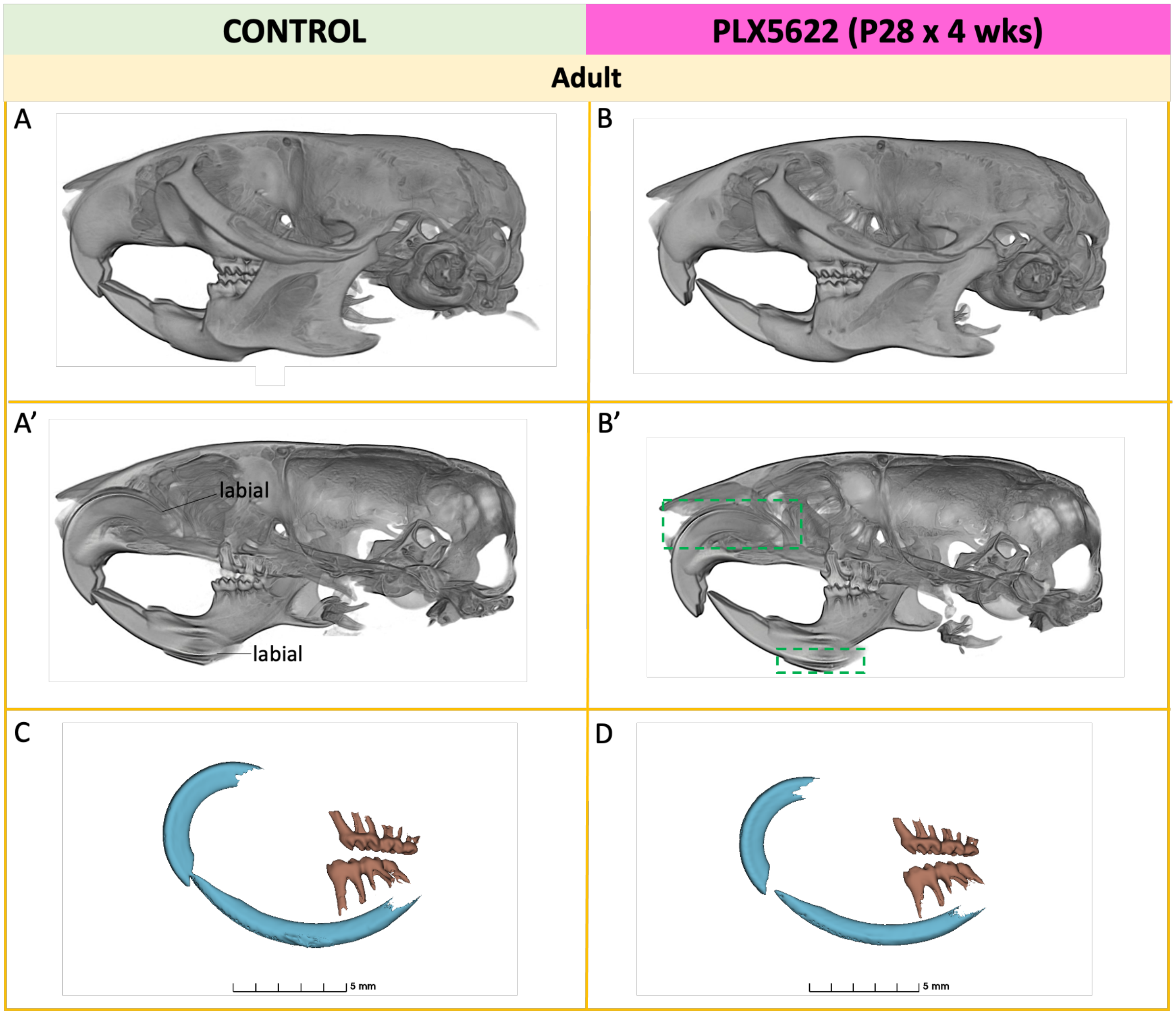

Figure S6. Inhibition of CSF1R in adult mice. µCT images of adult mice (P56) which were fed control (**A, A', C**) or PLX5622 (**B, B', D**) diets for four weeks, starting at P28. **A'** and **B'** are sagittal-cut section of 3D reconstructions of **A** and **B** respectively. The ability for continuous incisor elongation during adulthood is not compromised in CSF1R-inhibited mice (**B', D**). Similar to controls **(A', C**) maxillary and mandibular teeth properly occlude and exhibit no changes in shape (**B', D**). Newly added enamel on labial surfaces of maxillary and mandibular incisors appear normal (**A' v/s B'**, green boxes). *n* = 3 animals per group. [See Supplemental Table 1 for details].
